# Factors associated with dental interns and professors perception of the management of disabled patients: A cross-sectional study under multivariable analysis

**DOI:** 10.1016/j.heliyon.2024.e24213

**Published:** 2024-01-09

**Authors:** Natalia Gómez-Vilcapoma, Gissela Briceño-Vergel, Nancy Córdova-Limaylla, Marysela Ladera-Castañeda, Luis Cervantes-Ganoza, Clifford Allen-Revoredo, Miriam Castro-Rojas, César Cayo-Rojas

**Affiliations:** a Universidad Privada San Juan Bautista, School of Stomatology, Lima, Peru; b Universidad Nacional Federico Villarreal, Postgraduate School, Research Team “Salud Pública – Salud Integral”, Lima, Peru; c Universidad Inca Garcilaso de la Vega, Faculty of Stomatology, Lima, Peru

**Keywords:** Perception scale, Dentistry, Disability, Dental care, Cross-sectional study, Dental professors, Dental interns, Peru

## Abstract

**Background:**

Patients with disabilities experience oral health inequalities, including increased disease prevalence and unmet healthcare needs. The aim of this study was to assess factors associated to the perceived management of patients with physical disabilities among dental interns and professors at a university located in the capital city and a branch in a province in Peru.

**Methods:**

This cross-sectional, observational, and analytical study included 100 dental interns and 75 Peruvian dental professors and was conducted from January to April 2022. A validated perception scale was used to evaluate the management of disabled patients. For the statistical analysis, the study employed Pearson's chi-square test and Fisher's exact test, along with a Poisson regression model that used robust variance. The adjusted prevalence ratio (APR) was utilized to evaluate perception while taking into account factors such as gender, age, marital status, origin, professional experience, previous treatment of a patient with physical disability, and previous cohabitation with a disabled patient. The significance level was set at p < 0.05.

**Results:**

The 86 % of dental interns and 88 % of dental professors had a poor perception of managing disabled patients, with no significant association between them (p = 0.698). Male and female dental interns displayed significant differences in perception (p = 0.004), while no other variables showed significant differences (p < 0.05). Conversely, dental professors displayed significant differences in all variables analyzed (p < 0.05). In dental interns, gender was found to be the only influential variable, with females having a 41 % higher likelihood of perceiving disabled patient management poorly compared to males (APR = 1.41; 95 % CI: 1.04–1.91) (p = 0.028). However, gender was not found to be a significant factor for dental professors (p = 0.449).

**Conclusion:**

The majority of dental interns and professors had a poor perception of managing disabled patients, with no significant differences observed between them. Moreover, gender significantly influenced the perception of managing patients with physical disabilities among dental interns specifically. On the other hand, neither age, marital status, origin, professional experience, previous treatment of a patient with physical disability, nor previous cohabitation with a disabled patient were found to be associated factors among dental interns and professors.

## 1 Introduction

Individuals with disabilities have permanent physical, mental, intellectual, or sensory impairments which can hinder their full and effective participation in society due to various obstacles and difficulties they face [1]. The social perception of disability has an impact on people' inclusion, requiring greater sensitivity, empathy and a better attitude towards the disabled [2].

Disability rates are on the rise globally due to a combination of factors, including increased life expectancy among children with disabilities, aging populations, and a growing incidence of chronic health issues. These trends are negatively impacting more than one billion individuals [3,4]. According to a report from the World Health Organization, approximately 15 % of the global population is living with some form of disability, with elderly individuals and women experiencing the highest rates [1,5]. This equates to roughly 110 to 190 million individuals worldwide who struggle with functional limitations. In Latin America and the Caribbean, the number of children with disabilities is estimated to be almost 19.1 million [3,4].

In Peru, the National Disability Observatory estimates that the population with disabilities will reach 1,737,865 in 2022, equaling approximately 5 % of the total population [6,7].

Individuals with disabilities exhibit a greater incidence of oral health problems when compared to the general population. According to reports from studies conducted in this population, dental caries prevalence rates were 45 % in the United States, 86.4 % in Turkey, 38 % in India, 51 % in Pakistan, and 42.4 % in Rwanda (Tefera and Purohit). As a result, oral pathologies in children with disabilities may be more pronounced. Poor oral hygiene has been linked to increased calculus deposition, a higher Community Periodontal Index (CPI), and a higher prevalence of caries and malocclusion, according to multiple studies [[8], [9], [10]]. Despite the negative biological, psychosocial, and emotional impacts of oral and systemic diseases, people often prioritize their physical health over their oral health [5]. Patients with disabilities face even greater challenges due to limited access to health services, as the National Institute of Statistics and Informatics (INEI) has reported that 22.7 % of the Peruvian population with disabilities lack health insurance [11,12]. According to the World Health Organization (WHO), individuals with disabilities tend to receive the least attention in dental healthcare services, primarily due to various barriers. These obstacles include dental practitioners' inexperience leading to insecurity in care, possible ergonomic limitations stemming from inadequate dental infrastructure conditioning, a deficiency in skills for proper interpersonal communication, or a lack of knowledge among family caregivers regarding the significance of maintaining good oral hygiene. As a result, individuals with disabilities have a higher likelihood of developing oral diseases [7,[13], [14], [15], [16], [17]].

Some studies have suggested that dental schools may not be adequately preparing students to care for patients with disabilities. To address this issue, experts recommend that curricula include topics that guide students in the appropriate clinical care of these patients using a holistic approach [[18], [19], [20]]. In addition, it is suggested that professors who teach these topics should have appropriate training [5,19,20].

Some studies have discovered a significant connection between educational level and years of clinical experience regarding treatment perception for patients with disabilities [5,21]. However, expertise in previously treating this type of patient was found to be significantly associated, while gender, educational level, and years of experience showed no significant association [17].

Individuals with disabilities experience disparities in oral health, such as higher disease rates and unaddressed health concerns [18]. Thus, during dental education, it is crucial to teach students about promoting and preventing strategies which help them acquire the knowledge and abilities necessary to provide efficient treatment to those with special needs, taking into account their limitations or disabilities [15]. In this context, it is crucial for dental students and professionals to possess sensitivity, knowledge, and the development of soft skills when approaching patients. This will enable them to provide high-quality, individualized care leading to successful outcomes [15,22,23].

To improve management of disabled patients during dental care, it is crucial to understand the perspectives of dental interns and professors. Establishing strategies and guidelines based on their perceptions can help minimize barriers [5].

Therefore, the aim of this study was to assess factors associated to the perceived management of patients with physical disabilities among dental interns and professors at a university located in the capital city and a branch in a province in Peru.

## 2 Materials and methods

### 2.1 Type of study and delimitation

This cross-sectional design study was conducted from January to April 2022 on dental interns and professors at Universidad Privada San Juan Bautista (UPSJB). The university has a campus in Lima, the capital, and another branch in Ica, a Peruvian province. The manuscript was written in compliance with the STrengthening the Reporting of OBservational studies in Epidemiology (STROBE) guidelines for observational research [24].

### 2.2 Population and selection of participants

The study population included a total of 189 participants, comprising of 108 dental interns and 81 dental professors. The sample size did not need to be calculated as the entire target population (N = 175) of 100 dental interns and 75 dental professors met the eligibility criteria and were included.

### 2.3 Inclusion criteria

- Dental interns enrolled in the first semester of the 2022 academic year.
- Permanent dental professors at UPSJB.
- Dental professors recruited at UPSJB during 2022-1.

### 2.4 Exclusion criteria

- Dental interns and professors who did not complete the scale.
- Dental interns and professors who abandoned the academic period.
- Dental interns and professors with a physical disability.

### 2.5 Variables

The variable of interest was perception on the management of disabled patients, and the variables of association were gender, age, marital status, origin, professional experience, previous treatment of a patient with physical disability, and previous cohabitation with a disabled patient. Physical disability that causes some kind of impairment in activities of daily living was considered.

### 2.6 Instrument application

The validated scale employed was focused on the management of patients with disabilities [5]. It included 17 items assessing perception of the management of disabled patients. A bilingual professional translated the content into Spanish and tailored it to the local culture. Three experts in dental research validated the items for their consistency, sufficiency, relevance, timeliness, objectivity, clarity, and organization, resulting in an Aiken V validity coefficient of 0.84 (95 % CI: 0.79–0.87), which was considered acceptable. Each of these items had five alternatives on an ordinal scale (Likert type) from "Strongly disagree" (1 point) to "Strongly agree" (5 points). For negative items such as R6, R7, R8, and R9, the participants' scores were reversed from "Strongly agree" (1 point) to "Strongly disagree" (5 points). The combined scores of dental interns and professors were used to determine participants' perception levels. Those scoring between 17 and 59 points were deemed to have poor perception, while those scoring between 60 and 85 points were classified as having good perception. The instrument's cut-off point was established by averaging the full score of the indifferent (51 points) and agree (68 points) categories, resulting in 59.5 points. The precision of the cut-off point was verified through Livingston's K^2^ coefficient, yielding acceptable values of 0.864 and 0.888 for the dental interns and professors, respectively.

To assess the instrument's reliability, we utilized Cronbach's alpha and obtained satisfactory values for dental interns (0.79; 95 % CI: 0.72–0.84) and professors (0.76; 95 % CI: 0.67–0.83). Moreover, we administered the scale to 30 randomly chosen participants from each group twice in a 7-day interval, with the questions' order altered to circumvent recall bias (test-retest) [25,26]. The Spearman's correlation coefficient results were positive for both dental interns (0.89; 95 % CI: 0.77–0.95) and professors (0.90; 95 % CI 0.79–0.95).

### 2.7 Procedure

The scales were developed in Google Classroom® and distributed asynchronously with the permission of the Professional School Management to dental interns and professors. The link was sent directly to their institutional emails and through the WhatsApp® and Facebook® social networks. To participate in the study, an informed consent form was provided at the beginning of the scale, followed by instructions for its development. However, participants were given the option to decline the evaluation if they preferred not to complete it during the assessment. To prevent duplicate responses, the online questionnaire was designed to allow only one submission per corresponding email. The data was accessed only by the researchers, and no personal information such as name, phone number, or address was collected. Only one submission was taken into account for each dental intern and professor. After completion of the study, the results were emailed to the individuals who requested them at their respective personal email addresses. No incentives were offered to the invitees in exchange for their participation in the survey, which was accessible from January 6th to April 30th, 2022.

### 2.8 Statistical analysis

Data analysis was conducted using version 28.0 of the Statistical Package for the Social Sciences (SPSS) (IBM, Armonk, New York, USA). Relative and absolute frequencies were calculated by utilizing descriptive statistics. Bivariate analysis was performed through Pearson's chi-square test and Fisher's exact test (for expected values less than 5). Additionally, a Poisson regression model with robust variance was developed using the adjusted prevalence ratio (APR) to evaluate potential influential variables. The significance level set was p < 0.05.

## 3 Ethical issues

All participants provided informed consent for this study. The study adhered to bioethical principles for medical research involving human subjects as outlined in the Declaration of Helsinki [27], including confidentiality, freedom, respect, and non-maleficence. The data were securely stored on a password-protected device accessible only to the researchers. The Institutional Ethics Committee of the Universidad Privada San Juan Bautista approved the present study with Resolution No. 1386-2021-CIEI-UPSJB.

## 4 Results

The response rates for dental interns and professors were 96.15 % and 94.93 %, respectively. Of the 175 participants, the mean age for 100 dental interns was 26.6 ± 5.1 years, and that for 75 dental professors was 42.2 ± 8.9 years. The primary gender for both groups was female, with 79 % of interns and 66.7 % of professors. The majority of dental interns were single (88 %), while most dental professors were married (56.0 %). 62 % of dental interns lived in the capital city while 39 % of dental professors lived in the province. Finally, it was found that 76 % of dental professors possessed professional experience of 10 years or more [Table 1].

**Table 1:** Characterization of the sociodemographic variables of dental interns and professors.

Variables	Categories	Dental interns	Dental professors	Total
		f (%)	f (%)	f (%)
Gender	Female	79 (79.0)	50 (66.7)	129 (73.7)
	Male	21 (21.0)	25 (33.3)	46 (26.3)
^(a)^ Age group	<26 years old	52 (52.0)		52 (52.0)
	≥26 years old	48 (48.0)		48 (48.0)
	<42 years old		38 (50.7)	38 (50.7)
	≥42 years old		37 (49.3)	37 (49.3)
Marital status	Single	88 (88.0)	33 (44.0)	121 (69.1)
	Married or cohabiting	12 (12.0)	42 (56.0)	54 (30.9)
Origin	Capital city	62 (62.0)	36 (48.0)	98 (56.0)
	Province	38 (38.0)	39 (52.0)	77 (44.0)
Professional experience	<10 years		18 (24.0)	18 (24.0)
	≥10 years		57 (76.0)	57 (76.0)
Have you ever treated a patient with a physical disability?	Yes	48 (48.0)	61 (81.3)	109 (62.3)
	No	52 (52.0)	14 (18.7)	66 (37.7)
Do you live with or have you ever lived with someone who had any type of physical disability?	Yes	33 (33.0)	14 (18.7)	47 (26.9)
	No	67 (67.0)	61 (81.3)	128 (73.1)
**Age**	**Mean ± SD**	26.6 ± 5.1	42.2 ± 8.9	33.3 ± 10.4

(a): Cut-off point in the means for each group; f: absolute frequency, SD: Standard Deviation.

On the other hand, 48 % of dental interns and 81.3 % of dental professors have reported treating a patient with a physical disability. Additionally, only 33 % of residents and 18.7 % of faculty have experience living with someone with a physical disability [Table 1].

No significant association was found between the perception of disabled patient management among dental interns and professors (p = 0.698). Furthermore, 86 % of dental interns and 88 % of dental professors had poor perception on the management of disabled patients. On the other hand, when comparing the total perception scores of dental interns and professors, it was observed that there were no significant differences (p = 0.793) [Table 2].

**Table 2:** Association of the perception on the management of disabled patients by dental interns and professors.

Participants	f (%)	Perception		χ^2^	*p	Total Perception Score		
		Poor	Good			Mean (SD)	Median (IQR)	**p
**Dental interns**	100 (100.0)	86 (86.0)	14 (14.0)	0.15	0.698	53.5 (8.1)	53.5 (6.0)	0.793
**Dental professors**	75 (100.0)	66 (88.0)	9 (12.0)			53.2 (5.9)	54.0 (8.0)	

f: absolute frequency; χ^2^: Pearson's chi-square, *p < 0.05 (significant differences). SD: Standard Deviation, IQR: InterQuartile Range, **Based on Mann Whitney *U* test as scores were not normally distributed (p < 0.05, significant differences).

Significant discrepancies were found between male and female dental interns (p = 0.004) regarding their perceptions of managing disabled patients. However, there were no significant differences with respect to age (p = 0.321), marital status (p = 1.000), origin (p = 0.433), whether or not they had treated a disabled patient before (p = 0.321) and whether or not they lived with someone who had a physical disability (p = 0.590) [Table 3].

**Table 3:** Perception on the management of disabled patients associated with sociodemographic factors of dental interns and professors.

Variable	Categories	Perception of dental students		*p	Perception of dental professors		p
		Poor	Good		Poor	Good	
		f (%)	f (%)		f (%)	f (%)	
**Gender**	Female	72 (91.1)	7 (8.9)	0.004*	45 (90.0)	5 (10.0)	0.451
	Male	14 (66.7)	7 (33.3)		21 (84.0)	4 (16.0)	
**Age group**	<26 years old	43 (82.7)	9 (17.3)	0.321			
	≥26 years old	43 (89.6)	5 (10.4)				
	<42 years old				33 (86.8)	5 (13.2)	1.000
	≥42 years old				33 (89.2)	4 (10.8)	
**Marital status**	Single	76 (86.4)	12 (13.6)	1.000	31 (93.9)	2 (6.1)	0.296
	Married or cohabiting	10 (83.3)	2 (16.7)		35 (83.3)	7 (16.7)	
**Origin**	Capital city	52 (83.9)	10 (16.1)	0.433	32 (88.9)	4 (11.1)	1.000
	Province	34 (89.5)	4 (10.5)		34 (87.2)	5 (12.8)	
**Professional experience**	non-professional	86 (86.0)	14 (14.0)				
	<10 years				16 (88.9)	2 (11.1)	0.915
	≥10 years				48 (84.2)	9 (15.8)	
**Have you ever treated a physically disabled patient?**	Yes	43 (89.6)	5 (10.4)	0.321	54 (88.5)	7 (11.5)	1.000
	No	43 (82.7)	9 (17.3)		12 (85.7)	2 (14.3)	
**Do you live with or have you ever lived with someone who had any type of physical disability?**	Yes	27 (81.8)	6 (18.2)	0.590	12 (85.7)	2 (14.3)	1.000
	No	59 (88.1)	8 (11.9)		54 (88.5)	7 (11.5)	

*Based on Pearson's chi-square, p < 0.05 (significant differences).

No significant differences were found among dental professors in relation to the variables of gender (p = 0.451), age (p = 1.000), marital status (p = 0.296), origin (p = 1.000), professional experience (p = 0.915), whether or not they had treated a disabled patient before (p = 1.000) and whether or not they lived with someone who had a physical disability (p = 1.000) [Table 3].

Based on the multivariable regression model, gender was the only significant variable in dental interns, with females having a 41 % higher likelihood of perceiving poor management of disabled patients (APR = 1.41; 95 % CI: 1.04–1.91) (p = 0.028) compared to males. On the other hand, in dental professors, gender was not a significant factor (p = 0.449). However, age, marital status, origin, professional experience, previous treatment of a patient with physical disability, and living with this type of patient did not demonstrate significant impact on dental interns (p > 0.05) or professors (p > 0.05) **[**Table 4**].**

**Table 4:** Multivariable regression model of perception on the management of disabled patients in dental interns and professors according to associated factors.

Variable	Categories	Crude model								Adjusted model			
		Perception of dental interns				Perception of dental professors				Perception of dental interns			
		p*	PR	95 % CI		p*	PR	95 % CI		p**	APR	95 % CI	
				LL	UL			LL	UL			LL	UL
**Gender**	Female	0.048*	1.37	1.00	1.86	0.449	1.09	0.88	1.35	0.028**	1.41	1.04	1.91
	Male		1.00				1.00				1.00		
**Age group**	<26 years old	0.319	0.92	0.79	1.08					0.140	0.89	0.76	1.04
	≥26 years old		1.00								1.00		
	<42 years old					0.353	0.92	0.76	1.10				
	≥42 years old						1.00						
**Marital status**	Single	0.793	1.04	0.79	1.35	0.574	1.06	0.88	1.27	0.275	1.16	0.89	1.51
	Married or cohabiting		1.00				1.00				1.00		
**Origin**	Capital city	0.411	0.94	0.80	1.09	0.855	1.02	0.84	1.23	0.519	0.95	0.82	1.10
	Province		1.00				1.00				1.00		
**Professional Experience**	<10 years					0.593	1.06	0.87	1.29				
	≥10 years						1.00						
**Have you ever treated a physically disabled patient?**	Yes	0.319	1.08	0.93	1.27	0.497	1.11	0.83	1.49	0.173	1.11	0.96	1.29
	No		1.00				1.00				1.00		
**Have you ever lived with or currently live with an individual who has a physical disability?**	Yes	0.432	0.93	0.77	1.12	0.964	1.01	0.79	1.28	0.330	0.92	0.77	1.09
	No		1.00				1.00				1.00		

PR: Prevalence Ratio; APR: Adjusted Prevalence Ratio; *Based on simple Poisson regression with robust variance (Crude model); **Based on Poisson multiple regression with robust variance (Adjusted model); 95 % CI: 95 % confidence interval. Significant association (p < 0.05).

When analyzing the Perception Scale items regarding the management of disabled patients among dental intern students in relation to gender, statistically significant associations were found with R5 (I consider it a complex situation to provide dental treatment to a physically disabled patient), R6 (I prefer NOT to treat a patient with blindness because I consider it difficult), R8 (I prefer NOT to treat a patient with speech disability because I consider it difficult), R9 (I prefer NOT to treat a patient in a wheelchair because I consider it difficult) and R12 (I believe that my workplace has adequate facilities to care for a physically disabled patient) (p = 0.029, p = 0.039, p = 0.025, p = 0.008 and p = 0.047; respectively) **[**Table 5**].**

**Table 5:** Gender-associated perception scale on the management of disabled patients in dental interns.

Questionnaire		Strongly disagree	Disagree	Indifferent	Agree	Strongly agree	Gender
		f (%)	f (%)	f (%)	f (%)	f (%)	*p
**R1.** I believe I have adequate knowledge to care for any physically disabled patient.	**I**	0 (0.0)	17 (17.0)	7 (7.0)	63 (63.0)	13 (13.0)	0.133
	**P**	0 (0.0)	20 (26.7)	2 (2.7)	39 (52.0)	14 (18.7)	
**R2.** I believe that a physically disabled patient is treated fairly.	**I**	0 (0.0)	32 (32.0)	13 (7.0)	35 (35.0)	20 (20.0)	0.200
	**P**	0 (0.0)	31 (41.3)	1 (1.3)	27 (36.0)	16 (21.3)	
**R3.** I consider it important to provide quality care to the physically disabled patient.	**I**	6 (6.0)	1 (1.0)	1 (1.0)	22 (22.0)	70 (70.0)	0.705
	**P**	3 (4.0)	1 (1.3)	1 (1.3)	18 (24.0)	52 (69.3)	
**R4.** At some point I learned that there was a dental clinic that provided treatment to patients with physical disabilities.	**I**	14 (14.0)	22 (22.0)	29 (29.0)	28 (28.0)	7 (7.0)	0.255
	**P**	9 (12.0)	14 (18.7)	11 (14.7)	32 (42.7)	9 (12.0)	
**R5.** I consider it a complex situation to provide dental treatment to a physically disabled patient.	**I**	5 (5.0)	32 (32.0)	17 (17.0)	42 (42.0)	4 (4.0)	*0.029
	**P**	6 (8.0)	23 (30.7)	3 (4.0)	33 (44.0)	10 (13.3)	
**R6.** I prefer NOT to treat a patient with blindness because I consider it difficult.	**I**	0 (0.0)	92 (92.0)	3 (3.0)	3 (3.0)	2 (2.0)	*0.039
	**P**	0 (0.0)	69 (92.0)	4 (5.3)	2 (2.7)	0 (0.0)	
**R7.** I prefer NOT to treat a patient with hearing impairment because I consider it difficult.	**I**	38 (38.0)	50 (50.0)	5 (3.0)	5 (5.0)	2 (2.0)	0.077
	**P**	28 (38.7)	39 (52.0)	4 (5.3)	3 (4.0)	0 (0.0)	
**R8.** I prefer NOT to care for a patient with speech impairment because I consider it difficult.	**I**	37 (37.0)	51 (51.0)	6 (6.0)	4 (4.0)	2 (2.0)	*0.025
	**P**	28 (37.3)	40 (53.3)	3 (4.0)	3 (4.0)	1 (1.3)	
**R9.** I prefer NOT to care for a patient in a wheelchair because I consider it difficult.	**I**	39 (39.0)	49 (49.0)	7 (7.0)	3 (3.0)	2 (2.0)	*0.008
	**P**	30 (40.0)	36 (48.0)	5 (6.7)	4 (5.3)	0 (0.0)	
**R10.** I feel that a physically disabled patient needs a dentist with special skills to be treated.	**I**	13 (13.0)	26 (26.0)	13 (13.0)	35 (35.0)	13 (13.0)	0.750
	**P**	11 (14.7)	26 (34.7)	4 (5.3)	27 (36.0)	7 (9.3)	
**R11**. I feel that a physically disabled patient needs special dental equipment to be treated.	**I**	5 (5.0)	27 (27.0)	13 (13.0)	43 (43.0)	12 (12.0)	0.404
	**P**	10 (13.3)	23 (30.7)	4 (5.3)	30 (40.0)	8 (10.7)	
**R12.** I believe that my workplace has adequate facilities to care for a physically disabled patient.	**I**	17 (17.0)	30 (30.0)	23 (23.0)	27 (27.0)	3 (3.0)	*0.047
	**P**	10 (13.3)	27 (36.0)	2 (2.7)	26 (34.7)	10 (13.3)	
**R13.** I consider that university academic training I received is adequate for the care of patients with physical disabilities.	**I**	10 (10.0)	16 (16.0)	18 (18.0)	48 (48.0)	8 (8.0)	0.181
	**P**	10 (13.3)	30 (40.0)	5 (6.7)	22 (29.3)	8 (10.7)	
**R14.** I feel that the physically disabled patient should always be accompanied during dental care to facilitate communication and management of situations.	**I**	3 (3.0)	3 (3.0)	3 (3.0)	51 (51.0)	40 (40.0)	0.185
	**P**	3 (4.0)	2 (2.7)	1 (1.3)	31 (41.3)	38 (50.7)	
**R15.** If I have to care for a patient with a physical disability, I would do it in a schedule with no patients waiting.	**I**	3 (3.0)	11 (11.0)	8 (8.0)	55 (55.0)	23 (23.0)	0.913
	**P**	5 (6.7)	11 (14.7)	6 (8.0)	38 (50.7)	15 (20.0)	
**R16.** I think I need more specialized courses on managing physically disabled patients.	**I**	2 (2.0)	4 (4.0)	6 (6.0)	51 (51.0)	37 (37.0)	0.125
	**P**	0 (0.0)	2 (2.7)	5 (6.7)	42 (56.0)	26 (34.7)	
**R17.** I believe that using a software or phone application would help me communicate better with a physically disabled patient.	**I**	2 (2.0)	9 (9.0)	26 (26.0)	47 (47.0)	16 (16.0)	0.606
	**P**	1 (1.3)	7 (9.3)	14 (18.7)	36 (48.0)	17 (22.7)	

I: Intern, P: Professor. *Based on Fisher's exact test for dental interns' responses, p < 0.05 (significant association).

In regards to dental interns, the R4 item (At some point I learned that there was a dental clinic that provided treatment to patients with physical disabilities), as well as R12 and R17 (I believe that employing a software or phone application would help me communicate better with a physically disabled patient), yielded the highest percentage of indifferent responses among all items **[**Table 5**].**

## 5 Discussion

The FDI World Dental Federation (FDI) assists countries in understanding and promoting the inclusion of persons with disabilities in health programs to improve access and coverage, develop policy tools and strengthen policy-making capacity to meet their needs. The FDI advises conducting oral health risk assessments and offering oral health promotion technique training to dental professionals as a vital component of overall health [28]. In light of the above, aim of this study was to assess factors associated to the perceived management of patients with physical disabilities among dental interns and professors at a university located in the capital city and a branch in a province in Peru.

Regarding the query "Have you ever treated a patient with a physical disability?", the study revealed that 81.3 % of dental professors had provided treatment to patients with physical disabilities. These results are in line with those reported by Crocé et al. (95.7 %) and Conciecao et al. (73.1 %), where professionals also treated physically disabled patients as part of their clinical work [29,30]. However, these findings contrast with those of Ajwa et al. who reported that 46.4 % of dental practitioners [5] have treated patients with physical disabilities. This difference in findings may be attributed to the fact that the previous study utilized a broad measurement encompassing individuals at various levels of education, including dental interns, dental clinic students, general dentists, specialists, and consultants. In contrast, the current study separated the results by dental interns and professors. About dental interns, 48.0 % reported treating a physically disabled patient, which corresponds to the outcomes of the study by Conciecao et al. revealing that 45.2 % attended to patients with similar characteristics [30]. Increased exposure to disabled patients during undergraduate studies is necessary to familiarize students, strengthen their knowledge, improve their perception, and demystify concepts about the type of care provided to these patients. Experiences and relationships throughout their academic training are critical to defining their professional profile [31,32].

The present study also revealed that dental interns and professors had a poor perception of the treatment of disabled patients, with 86 % and 88 %, respectively. This may be because the dental curricula of various universities in Peru lack specific subjects on caring for patients with disabilities [33]. Some courses only cover a few related topics. This is consistent with findings from studies by Salah et al. [34] and Ajwa et al. [5], who suggest that university education is insufficient for treating patients with disabilities. In addition, it is possible that other factors contribute to the negative perception uncovered in this study, including the length of time required to treat these patients, inadequate communication capabilities, patient non-cooperation, fear of harming the patient, and other possible variables [34].

In relation to the management of disabled patients associated with gender, the present study yielded that female dental interns had a significantly poor perception as compared to their male counterparts.

This was confirmed by the results of a multivariable regression model which showed that gender was the only influential variable, with women being 41 % more likely to have poor perception than men. These results are similar to those obtained by Mackensi et al. who reported that women felt less prepared and less comfortable handling patients with special needs than men [35]. This may be because female dental interns felt that caring for these types of patients could be more complex than they were prepared for. Consequently, a significant correlation was observed between gender and declining to care for patients with speech disabilities, blindness, or those in wheelchairs, given the complexity of the situation, with women exhibiting a higher percentage of poor perception. This finding is consistent with studies by Farooq et al. y Holtzman et al. who reported that women were the most sensitive to certain situations [36,37]. Another possible explanation for our findings regarding female dental interns could be neuroticism (trait of being anxious and emotionally vulnerable), as this is more common in women [38]. On the other hand, gender was not considered to be an influential factor in dental professors. This may be attributed to the regular training that professors receive as part of their professional development to enhance their expertise in the clinical management of diverse patient populations. Such training could aid in mitigating gender disparities in healthcare provision for disabled individuals [39].

Babik et al. noted the significance of family in influencing children's beliefs and attitudes towards others [40]. In the present study, dental interns and professors living with a physically disabled family member did not exhibit substantial differences in their perceptions compared to those without a disabled family member. These results contrast with those of Conciencao et al. [30], who found that having a disabled family member or friend affects an individual's behavior. This discrepancy may stem from the fact that Conciencao et al.'s [30] study was conducted in a distinct setting. It is known that social attitudes and intergroup biases are fostered within a dynamic cultural context, with particular demonstrations in the disability domain, given that disability is a socially constructed concept [40].

Previous experience in providing dental care to disabled patients did not improved the perception of dental residents and professors compared to those without such experience. This is probably due to the fact that during professional training it is necessary to develop educational interventions with real situations sustainable over time, in charge of specialized personnel to improve the emotional willingness, theoretical knowledge and clinical skills of dentists when facing disabled patients [41,42].

The importance of this study lies in highlighting the need to train dentists in the management of the health care of disabled patients and to enforce the mandatory inclusion of theoretical and practical subjects in the curricula, which will promote confidence, safety, empathy development and improvement of clinical skills in students, thus reducing barriers in caring for disabled patients [42]. Furthermore, these patients face limitations in expressing their needs and often struggle with self-care, resulting in an elevated risk for the prevalence and severity of oral cavity conditions [14,16]. The current study presents a distinct viewpoint from prior research [5,31,35] by elucidating the perceptions of both dental interns and professors towards caring for disabled patients. This holds relevance as it emphasizes the need for professors to possess suitable competencies to communicate knowledge, attitudes, and abilities to interns, thereby enhancing their clinical management skills in providing adequate care to these patients. The attainment of an inclusive quality education [43] is contingent upon the accomplishment of this objective. Additionally, our findings reveal an intriguing insight: while a significant cohort of dental interns and professors have observed dental care for disabled patients in the past, this does not enhance their perception compared to those who lack such experiences. Furthermore, our study suggests that living with disabled patients does not lead to an improvement in dental interns' and professors' perceptions regarding dental care.

One strength of the current study is that it investigates a topic that has undergone little research, particularly in Latin America [31,44]. This is significant as individuals with disabilities make up a vulnerable population that is often disregarded by various governments, particularly in countries where policies for these individuals are in the midst of structural adaptation [32].

Due to COVID-19 pandemic regulations, the study was limited by the inability to conduct in-person assessments. Additionally, the cross-sectional study design did not allow for dynamic assessments of the sustainability of perceptions toward management of disabled patients over time, and the correlation between dental intern perceptions and disabled patient satisfaction with care provided could not be determined. Another important limitation of this research is its narrow scope, as it was conducted exclusively in one Peruvian university located in the capital and only one province within Peru. For future studies aiming to generalize the obtained results, it would be recommended to extend the scope and include other cities in Peru as well as other countries.

The present study included variables that have been identified in existing literature as associated factors [5,17,21] in order to provide a comprehensive overview of the characteristics that impact dental interns' and dental professors' perceptions of dental care for patients with disabilities. The results of this study will help in the development of relevant strategies to reduce disparities. However, future studies should aim to replicate this research by using a series of open-ended questions to identify emerging variables and investigate ways to enhance dental care accessibility for patients with disabilities.

Given the cross-sectional nature of the present study, it is advisable to increase the sample size and examine whether gender still has a role to play in the negative perception of disabled patients' management. Furthermore, conducting an interview with disabled patients to understand their views on the treatment and conduct of dentists concerning their healthcare needs is recommended.

Academic dental programs should integrate multiple resources and/or pedagogical strategies into their curricula to foster attitudinal competencies among dental students. This will enable them to shape and cultivate their innate empathy [45], and subsequently deliver quality service to society in their professional lives, particularly to patients hindered by physical limitations [[46], [47], [48], [49], [50]]. Adapting the infrastructure is necessary to ensure ergonomic care for disabled patients [51]. Additionally, research should continue to improve the quality of life for patients with disabilities [7]. Finally, dental career curriculums must include courses fostering the acquisition of skills and competencies, such as reflective clinical practice, and provision of comprehensive dental services to people with disabilities [52,53].

## 6 Conclusion

In summary, despite the limitations of the present cross-sectional study, the perception of the majority of dental interns and professors regarding the management of disabled patients was poor, with no significant differences between them. Gender was a significant factor in dental interns' poor perception, with women having five times higher chances of holding a negative view of dental care for disabled patients compared to men. While a substantial group of participants reported previous experience in treating disabled patients, their perceptions were no better than those who did not report such previous experience. Living with disabled individuals did not enhance the perception of dental care. Age, marital status, origin, and professional experience were not found to be influential factors.

## CRediT authorship contribution statement

**Natalia Gómez-Vilcapoma:** Writing – review & editing, Writing – original draft, Project administration, Investigation, Conceptualization. **Gissela Briceño-Vergel:** Writing – review & editing, Writing – original draft, Project administration, Methodology, Investigation, Conceptualization. **Nancy Córdova-Limaylla:** Writing – review & editing, Validation, Methodology, Investigation. **Marysela Ladera-Castañeda:** Writing – review & editing, Validation, Methodology, Investigation. **Luis Cervantes-Ganoza:** Writing – review & editing, Methodology, Investigation. **Clifford Allen-Revoredo:** Writing – review & editing, Writing – original draft, Resources, Investigation. **Miriam Castro-Rojas:** Writing – review & editing, Resources, Methodology, Investigation. **César Cayo-Rojas:** Writing – review & editing, Validation, Supervision, Software, Investigation, Formal analysis.

## Declaration of competing interest

The authors declare that they have no known competing financial interests or personal relationships that could have appeared to influence the work reported in this paper.
